# Putative Role of *MCT1* rs1049434 Polymorphism in High-Intensity Endurance Performance: Concept and Basis to Understand Possible Individualization Stimulus

**DOI:** 10.3390/sports9100143

**Published:** 2021-10-18

**Authors:** Xavier Ramírez de la Piscina-Viúdez, Jesús Álvarez-Herms, Diego A. Bonilla, Arkaitz Castañeda-Babarro, Jon Larruskain, Julen Díaz-Ramírez, Ildus I. Ahmetov, Alex Martínez-Ascensión, Richard B. Kreider, Adrián Odriozola-Martínez

**Affiliations:** 1 Sport Genomics Research Group, Department of Genetics, Physical Anthropology and Animal Physiology, Faculty of Science and Technology, University of the Basque Country (UPV/EHU), 48940 Leioa, Spain; xabier.ramirezpiscina@gmail.com (X.R.d.l.P.-V.); jesusalvarez80@hotmail.com (J.Á.-H.); dabonilla@dbss.pro (D.A.B.); jlarruskain@hotmail.com (J.L.); julen2692@gmail.com (J.D.-R.); 2 Research Division, Dynamical Business & Science Society—DBSS International SAS, Bogotá 110311, Colombia; 3 Research Group in Biochemistry and Molecular Biology, Universidad Distrital Francisco José de Caldas, Bogotá 110311, Colombia; 4 Sport Science Research Group, Faculty of Psychology and Education, University of Deusto, 48007 Bilbao, Spain; arkaitzcb@gmail.com; 5 Department of Physical Education, Plekhanov Russian University of Economics, 117997 Moscow, Russia; genoterra@mail.ru; 6 Laboratory of Molecular Genetics, Kazan State Medical University, 420012 Kazan, Russia; 7 Research Institute for Sport and Exercise Sciences, Liverpool John Moores University, Liverpool L3 3AF, UK; 8 Computational Biology and Systems Biomedicine Department, Biodonostia Health Research Institute, P/Doctor Begiristain, 20014 Donostia, Spain; alexmaras1995@gmail.com; 9 Exercise & Sport Nutrition Laboratory, Human Clinical Research Facility, Texas A&M University, College Station, TX 77843, USA; rbkreider@tamu.edu; 10 kDNA Genomics^®^, Joxe Mari Korta Research Center, University of the Basque Country UPV/EHU, 20018 Donostia-San Sebastián, Spain

**Keywords:** lactate, genetics, endurance, metabolism, musculoskeletal, performance

## Abstract

Monocarboxylate transporters (MCTs) have been proposed as important mediators of the exchange between lactate (La^−^) producer and La^−^ recipient (consumer) cells. Previous studies have suggested that the *MCT1* A1470T genotype could be related to different physical performance phenotypes. This study followed the guidelines for Strengthening the Reporting of Genetic Association Studies (STREGA) and aimed to evaluate the distribution of the *MCT1* polymorphism rs1049434 in endurance-trained athletes compared to the untrained population. Moreover, this study explored the potential influence of the polymorphism alleles phenotypes on high-intensity exercise performance. In a cross-sectional study fashion, a total of 85 triathletes from northern Spain were genotyped for *MCT1* rs1049434 and compared to a control group of 107 healthy male participants (1000 Genomes Research Study for Iberian Populations in Spain). All athletes performed a 30 s Wingate all-out test (WAnT) on a cycle ergometer. Peak and mean power (absolute and relative) were measured. After verification of the Hardy–Weinberg equilibrium, the findings indicated that the *MCT1* TT genotype was overrepresented in triathletes in comparison to the genotypic frequency of the general Spanish population. No significant associations were found between any *MCT1* genotype and peak or mean power performance in the WAnT. Further studies are required to understand the relationship among *MCT1* A1470T polymorphism, endurance-trained athletes, and high-intensity performance.

## 1. Introduction

Lactate (La^−^) is undoubtedly one of the most studied metabolic markers measured in health, disease, and exercise sciences [1]. In regard to its metabolism, during stressful conditions such as maximal exercise, La^−^ acts as an energy source and has an important role within the acid–base regulation mechanisms, in addition to its action in several adaptive responses to the physical effort due to its hormone-like effects (“lactormone”) [2]. According to Brooks (2018), oxygen availability has been classically considered to be a primary driver of La^−^ production, although there are numerous reasons to reconsider this simplistic view [3]. For all-out (‘maximal’) explosive, high-intensity, or endurance efforts, the La^−^ accumulation is consequence of a higher rate of production versus a lower rate of removal rather than the muscle “anaerobiosis” (under physiological conditions). In fact, contrary to common belief, research on this topic has shown that no true “anaerobic conditions” occur during an intense physical effort and, therefore, this would not be a direct cause of increased La^−^ formation [4]. 

**Figure 1:**
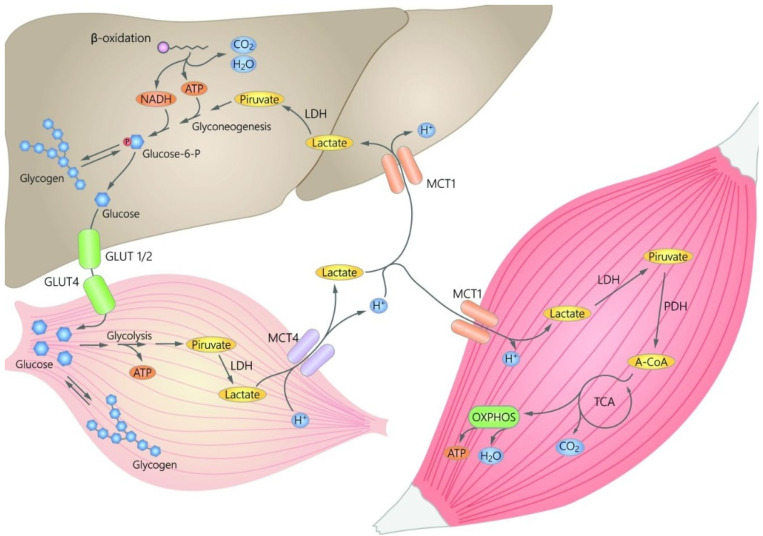
Schematic representation of the link among glycolysis, Cori’s cycle, and lactate oxidation complex proposed in the lactate shuttle hypothesis. This hypothesis explains the exchange between driver cells of lactate [La^−^] production and recipient cells of La^−^ consumption, which occurs within and among cells, tissues, and organs [3]. For physical exercise, fast-twitch muscle fibers (driver) produce lactate from glycolysis and express MCT4 at the sarcolemma for La^−^ export, whereas slow-twitch oxidative and fast-oxidative glycolytic fibers (consumers) express MCT1 in the sarcolemma and mitochondrial reticulum for La^−^ import and oxidation. On the other hand, some La^−^ travels through the bloodstream and is taken up in the liver, where it is converted back to glucose. LDH: lactate dehydrogenase, MCT4: protolinked monocarboxylate transporter isoform 4, MCT1: protolinked monocarboxylate transporter isoform 1, PDH: pyruvate dehydrogenase, TCA: tricarboxylic acid cycle, A-CoA: acetyl coenzyme A, OXPHOS: oxidative phosphorylation, NADH: reduced form of nicotinamide adenine dinucleotide, ATP: adenosine triphosphate. Source: designed by the authors (A.M.-A.) using the licensed version of Adobe Illustrator CC, 2017.

High-intensity exercise is characterized by the predominance of extramitochondrial pathways of energy production (i.e., phosphagen system and glycolysis), which drive the subsequent production and accumulation of hydrogen ions (H^+^), with the associated decrease in pH within fast-twitch muscle fibers [5]. It is this accumulation of H^+^ and other metabolites (Pi and poor Ca^2+^ handling) in the active skeletal muscle, and not the La^−^ concentration per se, which is linked to the acute reduction in force and power (muscle fatigue) [6,7]. Actually, due to the high energy requirements of the working muscle during physical exercise, a family of proton-linked symporters called monocarboxylate transporters (MCTs, also known as the SLC16A family) has been proposed as an important mediator of the exchange between La^−^ producer (driver) and La^−^ recipient (consumer) cells in intracellular, cell–cell, and tissue–tissue La^−^ shuttles in muscle, liver, heart, kidneys, and brain [2]. For example, La^−^ turnover involves the recycling process in the liver through Cori’s cycle, which provides glucose to the working skeletal muscle [8]. In particular, the MCT4 serves as the 1:1 transmembrane cotransport of La^−^ and H^+^ from muscle to the blood, while the MCT1 isoform is responsible for La^−^ uptake from the circulation. The latter enters the mitochondrial reticulum to support cell energy homeostasis via oxidative phosphorylation of ADP and creatine [3,9]. In this sense, during an endurance physical effort (where the predominant energy source is oxidative phosphorylation), La^−^ has shown an inhibitory effect on both lipolysis in adipose tissue (via certain G-coupled protein receptors, such as GPR81, and subsequent CREB activation) [10] and fatty-acid uptake in muscle (through the rise in malonyl-CoA, which inhibits carnitine palmitoyl transferase-1) [11]. Therefore, La^−^ may also regulate lipid oxidation and fuel utilization throughout exercise. Indeed, repeated La^−^ exposure and accumulation in active tissues from regular exercise results in adaptive processes such as mitochondrial biogenesis and improved metabolic flexibility [2]. Thus, the two isoforms of MCT1 and MCT4 are important La^−^/H^+^ cotransporters which are involved in the regulation of muscle pH and energy metabolism [12] (Figure 1).

It has been reported that the total La^−^ and H^+^ transport capacity is higher in slow-twitch oxidative muscle fibers (perhaps due to the greater MCT1 density) than in fast-twitch glycolytic muscle fibers [13]. Conversely, MCT4 density would be independent of fiber type and displays a significant interindividual variation, albeit related to the extramitochondrial metabolism capacity [14]. It has been shown that a single endurance exercise session (60% VO_2peak_ for 5–6 h) is able to increase the MCTs protein expression and to decrease muscle [La^−^] because of a higher transport and removal rate [15]. However, it seems that the expression of MCTs would depend on the type of physical effort, considering that an acute bout of high-intensity exercise (200% VO_2peak_ for 45 s) is associated with a significant decrease in both MCT1 and MCT4 relative abundance [16]. Consequently, the expression of both MCT isoforms can occur differently in response to a given stressor stimulus [3,14] but it seems that MCT1 protein expression is more sensitive to training than MCT4 [12]. 

The relative contribution to physical endurance performance of both MCT1 and MCT4 is not fully elucidated. Congenital disorders in *MCT1* have been found in patients with cryptic exercise intolerance, a rare condition in which individuals suffer severe chest pain and muscle cramping upon vigorous exercise [17]. This highlights the importance of MCT1 in response to exercise since it is related to changes in La^−^ metabolism (accumulation and clearance) after both strength/power [18] and endurance [5] exercise training. Recent studies suggest that a widespread single-nucleotide polymorphism (SNP) located in A1470T (rs1049434) of the *MCT1* gene is associated with different phenotypic profiles [19,20,21] and high athletic performance [22,23,24]. This common SNP results in a missense mutation A1470T that causes the change from aspartic acid to glutamic acid in codon 490 [19]. Due to the high frequency (30–50%) of this single mutation in the general population, it is no longer considered a pathogenic mutation but rather a non-disease-causing mutation [25]. Individuals with a minor (mutant) A allele have a 60–65% reduction in La^−^ transport rates and exhibit a higher [bLa^−^] concentration during a high-intensity effort [24]. This might be due to an impaired La^−^ transport from circulation to oxidative fibers in men carrying the A allele of *MCT1* rs1049434 [21]. In support of this, Fedotovskaya et al. [23] reported that the major T allele and the TT genotype of this polymorphism were significantly more prevalent in male Russian endurance athletes than in a control population. Alternatively, Sawczuk et al. [24] found that the MCT1 AA genotype was associated with elite sprint/power athletic status. These authors proposed that a higher [La^−^] in the muscle and blood of power-trained A allele carriers might be related to the hypertrophy response through regulation of mTORC1, IGF-1, and the growth hormone signaling pathways [26]. Kikuchi et al. [27] reported that the TT genotype of A1470T was overrepresented in Japanese wrestlers and associated with lower [bLa^−^] after a 30 s Wingate all-out test (WAnT) and during intermittent sprint tests.

Overall, previous studies have suggested that the *MCT1* A1470T genotype could be related to different physical performance phenotypes [25,27,28]. Accordingly, the *MCT1* T allele might be related to better endurance performance favoring La^−^ transport from blood to slow-twitch oxidative muscle fibers, consequently increasing the capability of using this La^−^ as a source of energy. Meanwhile, the A allele of the *MCT1* polymorphism might be associated with high-intensity performance via a better power training response. The aim of this study was to investigate the genotype distribution of the *MCT1* A1470T (rs1049434) polymorphism in endurance-trained northern Spanish athletes compared to the untrained population. Additionally, we explored the potential association between the *MCT1* A1470T polymorphism and the high-intensity performance measured by the 30 s WAnT. We hypothesized that genotype distribution would differ between athletes and general population, and that the *MCT1* A1470T polymorphism might influence high-intensity exercise performance in endurance-trained athletes.

## 2. Materials and Methods

### 2.1. Study Design

In a cross-sectional study fashion, the experimental protocol was double-blinded in the sense that neither the evaluators nor the participants knew the genotype during the physical test. The experimental procedures were conducted following the set of guiding principles for reporting the results of genetic association studies defined by the Strengthening the Reporting of Genetic Association studies (STREGA) guidelines, an extension of the STROBE statement [29].

### 2.2. Setting

All participants signed an informed consent form, which included (1) the goal of the study, (2) a statement for the unique use of the samples for this study, and (3) explicit anonymity regarding the final genetic result. The study protocol was approved by the Human Research Ethics Committee of the School of Science and Technology, University of the Basque Country (UPV/EHU) (M10_2017_108) in accordance with the Declaration of Helsinki and ethical standards in sport and exercise science research.

### 2.3. Subjects

Eighty-eight endurance-trained athletes (triathletes) from northern Spain volunteered and were eligible to participate in this study. The requirements to participate in the study were as follows: (a) conducting 10–14 h of physical exercise per week mainly based on endurance training and soft strength training, for more than 2 years, (b) refraining from carrying out any kind of regular power training, such as powerlifting, sprints, or short-distance fast running, and (c) being free of banned substances or doping penalties. Participants were neither treated nor hospitalized in the last 12 months. Participants that did not meet the inclusion criterion were excluded. Genotypic information of the general population (*n* = 107, control group) was obtained from data published in phase 3 of the 1000 Genomes Research Study for Iberian Population (Iberian Populations in Spain (IBS) available at https://www.internationalgenome.org/data-portal/population/IBS (accessed on 20 March 2020)) [30]. It is worth noting that different strategies have been implemented to protect the privacy of participants in genomic research projects; therefore, only genotypic information of the control group is available. For instance, the Ethical, Legal, and Social Implications (ELSI) group in the 1000 Genomes Project set up several anonymization practices to preserve privacy, mainly by oversampling and not collecting personal data other than sex [31].

### 2.4. Variables

Genotyping was considered as the main outcome (rs1049434 polymorphism for the *MCT1* gene). The body mass (BM, in kg), stature (cm), age (years), sex, sum of skinfolds (SF) in mm, and the 30 s WAnT were also measured and/or reported.

### 2.5. Data Sources/Measurement

#### 2.5.1. Anthropometry

The stature and BM were measured for each athlete following standard procedures. The SF thicknesses were measured in the triceps, subscapular, suprailiac, abdominal, mid-thigh, and medial calf using a Holtain Caliper (Holtain Ltd., Crymych, UK). All measurements were performed twice, and the results were averaged. The trained anthropometrist measured all participants according to the standard International Society for the Advancement of Kinanthropometry (ISAK) protocol. The intra-observer technical error of measurement was less than 7.5% for skinfolds and 1.5% for the other measurements, which is considered acceptable by the ISAK recommendations.

#### 2.5.2. MCT1 A1470T (rs1049434) Genotyping

We used buccal swabs (4N6FLOQSwab, Life Technologies, Carlsbad, CA, USA) to obtain saliva samples. Subsequently, the QIAmp DNA Mini Kit (Qiagen, Hilden, Germany) was used for DNA extraction, while quantification was carried out by fluorometry using Qubit (Life Technologies, Carlsbad, CA, USA) and the Quant-iT PicoGreen dsDNA Assay Kit (Invitrogen, Carlsbad, CA, USA). Finally, DNA samples were genotyped in the Biomark HD System (Fluidigm, South San Francisco, CA, USA) using an SNP type assay designed explicitly for rs1049434.

##### 2.5.3. 30 s Wingate All-Out Test (WAnT)

Endurance athletes performed a WAnT on an air-braked validated cycle ergometer (Wattbike Pro, Nottingham, UK) [18]. A rig was used for the dynamic calibration of the Wattbike ergometer based on first-principles approach by specialists at the Australian Institute of Sport [19]. During the 48 h preceding the test, participants refrained from any form of physical exertion that could influence the test results according to previous recommendations [20]. Prior to the test, all athletes performed a 10 min warmup on the cycle ergometer at 65% of their maximum heart rate, calculated according to the Karvonen protocol [21,22]. An S625X heart rate monitor (Polar Electro, Kempele, Finland) was used for measuring the heart rate. After warmup, a short rest period (120 s) was allowed while participants received instructions and prepared for the maximal (all-out) effort of the WAnT for 30 s [23]. The air-braking resistance was set to level 10, and the magnetic strength was set to level 1 (equating to 1045 W at 130 rpm and approximately 90–100 W increases for every further 5 rpm increase in cadence) [24]. The sprint test was carried out after 5 s of countdown being still. Athletes received verbal encouragement during the test, and the pedaling rate was recorded. The absolute and relative mean power (MP) and the peak power (PP) over the entire 30 s were calculated for each participant (per BM in kg).

#### 2.5.4. Statistical Analysis

Assessment of the Hardy–Weinberg equilibrium (HWE), a principle stating that the genetic variation in a population will remain constant from one generation to the next in the absence of perturbing factors, was carried out by means of the chi-square (χ^2^) test. Genotype distribution and allele frequencies between athletes and controls were compared using χ^2^ tests. The association of outcome variables (PP, MP, PP/BM, and MP/BM) with nongenetic factors (age, stature, BM, and the sum of SF) was investigated using linear regression. Moreover, the association of the *MCT1* A1470T polymorphism with each outcome variable was analyzed under dominant (TT + AT vs. AA), recessive (AA vs. AT + TT), and codominant (AA vs. AT vs. TT) models of inheritance. The whole-genome association analysis (WGassociation) function of the ‘SNPassoc’ package was used for this purpose, adjusting for the nongenetic variables significantly associated with each outcome variable. The false discovery rate method was used to correct the *p*-values of each outcome for multiple comparisons. All statistical analyses were performed using R version 3.2.3 (R Core Team 2015, R Foundation for Statistical Computing, Vienna, Austria) with the significance level set at *p* < 0.05.

## 3. Results

### 3.1. Participants

We evaluated and analyzed the results obtained from 85 endurance-trained males (age: 39.2 ± 7.9 years; body mass: 73.2 ± 7.2 kg; stature: 176.7 ± 5.9 cm). Three individuals were withdrawn by the researchers due to unsatisfactory genetic testing results. Figure 1 shows the selection, grouping, and final data analysis of the individuals in a flow diagram.

### 3.2. Outcome Data

The *MCT1* rs1049434 genotypes of endurance-trained athletes and controls exhibited an HWE distribution (*p* > 0.05). The frequency of the TT genotype was significantly higher in triathletes than in controls (*p* < 0.001) (Table 1).

**Figure 2:**
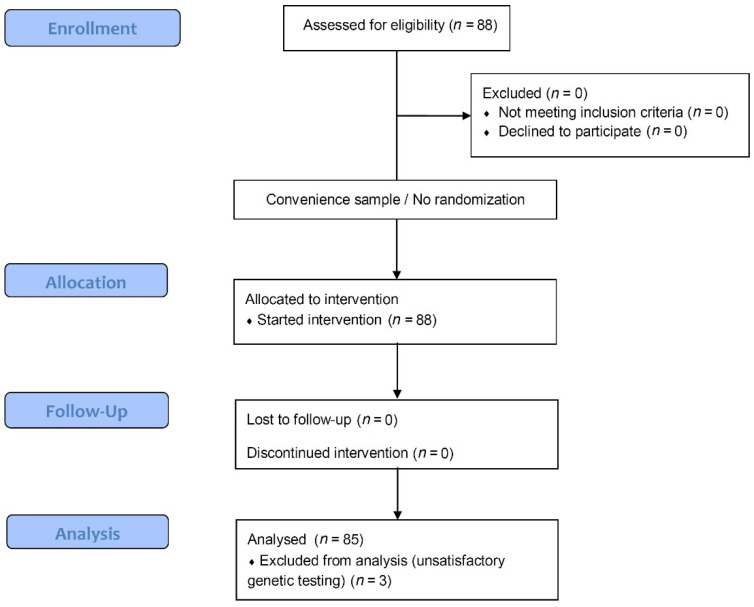
Flowchart of participant selection for the analysis.

The athletes were characterized according to different genotypes using dominant, recessive, and codominant models of inheritance, as shown in Table 2. Participants’ genotypes did not significantly alter the variance of stature, BM, or sum of SF; only the stature was significantly altered in the recessive model. The MP and PP values (absolute and relative) of the WAnT were not significantly different regarding *MCT1* rs1049434 genotypes (Figure 2).

## 4. Discussion

The first aim of the present study was to determine the possible differences in the *MCT1* A1470T genotype distribution between endurance-trained athletes and untrained population. Our findings showed that the *MCT1* TT genotype was overrepresented in endurance-trained athletes compared to the general population (nonathletes, i.e., controls) (*p* < 0.05). Although we did not measure [bLa^−^], our results are partially in concordance with Fedotovskaya et al. [23] considering that the TT genotype was predominant in Russian endurance athletes, who showed greater La^−^ clearance in comparison with other genotypes. In this study, La^−^ concentrations could not be measured due to technical issues. However, according to previous evidence [28,32], we can hypothesize that the T allele might be associated with benefits in increasing La^−^ transport from circulation to slow-twitch oxidative muscle fibers. Once there, La^−^ (not pyruvate) enters the mitochondrial reticulum to provide cell energy to resynthesize ATP and phosphocreatine via oxidative phosphorylation [2]. Kikuchi et al. [27] previously reported in wrestlers that the *MCT1* genotype is related to a higher power phenotype. In fact, the authors concluded that the TT genotype of the *MCT1* A1470T polymorphism was overrepresented in wrestlers compared with controls. These results are, to some extent, in concordance with the explanation proposed by Sawczuk et al. [24], who suggested that these possible genotype differences are related to a more reduced capability of La^−^ clearance in the AA genotype. This might cause a higher [La^−^] in the muscle and blood during high-intensity exercise. In fact, this phenomenon has been associated with the activation of power and hypertrophy signaling pathways such as mTORC1, IGF-1, and the growth hormone [26]. Two essential points should be noted here; both mentioned studies connected the La^−^ kinetics with the *MCT1* genotype, and they analyzed only strength/power athletes. The current knowledge regarding interindividual physiological and molecular allostatic response is limited; one could hypothesize that the TT genotype might be overrepresented in endurance athletes because they have a better “second shot” of La^−^ in oxidative muscle fibers [33]. 

Al-Haggar et al. [25] reported that different training stimuli possibly have a critical effect on epigenetic factors and the adaptive physiological functions of the *MCT1* polymorphism in the long term. This might explain why changes in MCT content are more common in response to chronic contractile activity [12]. This adaptive response of MCT1 content has been reported to occur mainly in the mitochondria when exercise training intensity is performed regularly above the VO_2max_ [33]. Thus, the high intensity and the regulation of internal pH would be of minor importance to improve physical performance under all-out conditions [2]. Under these premises, despite MCT1 content being a critical element involved in the regulation of cellular acidosis as an La^−^ transporter, their metabolic kinetics adaptations in the long-term after a specific and regular high-intensity stimuli could be more relevant (oxidative stress, inflammatory markers, miRNAs, etc.) than only the genotype [2].

The second aim of this study was to determine if there was a statistical association between the *MCT1* A1470T polymorphism and the high-intensity performance in nonpower-trained athletes. The PP and MP output during a WAnT indicated that participants of our study showed excellent fitness (see results in Table 2) according to available data from endurance-trained cyclists/triathletes [34,35]. Although the sample size could limit the detection of small genetic effects of this SNP on high-intensity endurance, no significant differences were found between the *MCT1* A1470T polymorphism and the measured physical variables. The study of genetic inheritance effects allows for estimation of the genetic contribution to total variance in a given performance variable [36]; thus, readers should note that models of inheritance allow exploring the biological rationale behind a given genotype. Similar to the present study, other works have linked *MCT1* genotypes to all-out/power performance. Massidda et al. [28] investigated the association between the *MCT1* A1470T polymorphism and different football phenotypes from five different countries. They reported that forwards (faster athletes) have a predominance of the TT genotype, this to frequent sprint training. Sawczuk et al. [24] also reported how elite sprint/power athletes were more likely than national-level athletes to have the AA genotype compared to TT. Lastly, TT genotypes might be more prone to suffer injury incidents than AA genotype, possibly related to the acidic intracellular environment [37]. Therefore, according to the results of our study, we suggest that the absence of differences in high-intensity endurance performance comparing genotypes for *MCT1* polymorphism could be caused by the characteristics of the athletes’ training routines more so than their maximal biological potential. The majority of athletes included in the present study followed a similar low-to-moderate intensity exercise program that does not include regular maximal stimuli, which is in agreement with the frequent training practices that are typically made up of more prolonged and slower-paced sessions [27]. However, endurance athletes may also use different training approaches that include high-intensity exercises; however, further research is needed to evaluate physiological adaptations in this regard.

Overall, the *MCT1* A1470T genotype could be a candidate to be analyzed in athletes leading to a better individualization of high-intensity endurance in response to power training. Notwithstanding, the perspective of categorize power performance phenotypes according to their *MCT1* genotype could be complemented by other physiological phenomena, such as those involving the microbiome status and the hepatic gluconeogenesis adaptive response. Therefore, to some extent, the La^−^ concentration after a standardized WAnT protocol does not explain per se the high-intensity endurance performance, but this potential explanation needs to be explored in the future with parallel evaluations of microbiome and short-chain fatty acid (SCFA) status in the colon. It is clear, however, that La^−^ is a chief messenger involved in a complex feedback loop with physiological implications, being more than only a metabolic waste product related to fatigue [3]. Thus, MCT transporters after different rates of La^−^ flux, oxidation, and gluconeogenesis are important not only as a biomarker describing the endurance performance accurately. The genotype might not only be essential to know the possible physiological potentialities, including endurance performance, but could also be essential to understand normal physiology through supplying fuel, maintaining glycemia and cerebral metabolism, and signaling [2].

### 4.1. Future Directions

Although the majority of studies regarding MCTs and exercise performance have focused mainly on La^−^ metabolism and its relationship with acidosis, no previous studies have explored the possible adaptive mechanisms involving individual phenotypes of microbiome status (SCFAs mainly in the colon). Thus, by connecting these conclusions with the above-described rationale of the microbiome (La^−^ regulation by SCFAs), one can hypothesize that AA genotypes may develop other adaptive physiological responses to mitigate this possible disadvantage related to greater acidosis in skeletal muscle. Indeed, MCT1 is not only important as a myocyte membrane transporter but also expressed in other tissues such as the gut epithelium to facilitate the absorption of SCFAs produced by gut microbiota [38]. In this regard, it has been previously reported that the bacterial microbiome could be able to regulate La^−^ homeostasis through the absorption and resynthesis of SCFAs, which are transported through the portal vein to the liver parenchyma promote gluconeogenesis [8].

The study of the gut microbiome has been related to La^−^ metabolism and endurance performance via La^−^-utilizing bacteria. For example, (1) the major pathway metabolizing La^−^ to propionate is at higher relative abundance post exercise; (2) La^−^ crosses the epithelial barrier into the lumen of the gut; (3) *Veillonella* relative abundance is increased in marathon runners post competition; (4) *Veillonela atypica* utilizes La^−^ as their sole carbon source producing propionate; (5) intrarectal instillation of *Veillonella atypica* or propionate in mice is sufficient to increased treadmill run time performance [39]. SCFAs show essential regulatory functions in metabolism, anti-inflammation, and the immune system [40], in addition to being transported and used as energy metabolites in different cell types, particularly by hepatocyte cells, which use propionate for gluconeogenesis. Since MCT1 is widely expressed in almost all human tissues, and its substrate selectivity includes additional endogenous metabolites such as acetate, propionate, and pyruvate in the colon [41], SCFAs generated by fermentation of dietary fiber by intestinal bacteria enter into colonic epithelial cells via MCT1 in order to serve as the major metabolic fuel for these cells under physiological conditions, particularly butyrate. This might represent a key mechanism for the influx/efflux across the polarized membrane of human intestinal epithelial cells [42]. Hence, it is possible that bacterial species (i.e., *Lactobacillus* spp.) influence exercise performance by producing La^−^, which in turn can be used by La^−^-utilizing bacteria to produce butyrate [43]. Furthermore, our research group has proposed a possible host genetics–microbiome interaction regarding GALNTL6 rs558129 endurance-related polymorphism and SCFA production by La^−^-consuming bacteria [44]. Thus, despite the MCT1 isoform acting directly on the La^−^ kinetics, the other metabolic pathways that use La^−^ as a substrate hinders the definitive conclusion of their implications in better/worse endurance performance. In summary, although the measurement of La^−^ response after maximal exercise is scientifically accepted and used as a specific biomarker of endurance performance, the limitations of their analysis are currently being discussed.

### 4.2. Limitations

The lack of [bLa^−^] measures in the present study hinders the establishment of a relationship between the *MCT1* A1470T genotype and metabolic processes. However, this was not the primary aim of the present study in which we directly measured all-out explosive exercise performance. In this regard, it could be interesting to measure [La^−^] after a WAnT in power-trained and nonpower-trained individuals. Furthermore, it could be valuable to perform more than one WAnT to measure La^−^ clearance and fatigue index between tests, in order to have a better understanding of the relationship between MCT1 A1470T genotype and [La^−^] kinetics in each case.

## 5. Conclusions

The *MCT1* TT genotype is overrepresented in Spanish endurance-trained athletes compared to the general population; however, no significant associations were found between any *MCT1* A1470T genotype and WAnT performance. Due to the multifactorial and polygenic nature of high-intensity endurance exercise, further studies are still necessary, including other genetic polymorphisms, environmental factors and epigenetic modifications, physiological factors related to individual adaptations (oxidative stress, inflammation, miRNAs), and/or the impact of La^−^-consuming organisms from the gut microbiota on La^−^ metabolism.

## Figures and Tables

**Table 1:** Comparison of genotype frequencies between triathletes and control group.

	Triathletes			Control		
	*n*	Frequencies (%)	*p*-Value	*n*	Frequencies (%)	*p*-Value
**T**/**T**	33	38.8	>0.05	26	24.3	>0.05
**A**/**T**	33	38.8		61	57.0	
**A**/**A**	19	22.4		20	18.7	

*MCT1* gene distributions were in Hardy–Weinberg equilibrium (*p* > 0.05).

**Table 2:** Results of WAnT across all genotypes using the codominant, dominant, and recessive allele models.

	Codominant Model				Dominant Model		Recessive Model	
	T/T	A/T	A/A	*p*	A/T + A/A	*p*	A/T + T/T	*p*
	(n = 33)	(n = 33)	(n = 19)		(n = 52)		(n = 66)	
Age (years)	40.3 ± 6.9	38.9 ± 8.1	37.8 ± 9.6	0.374	38.5 ± 8.6	0.171	39.6 ± 7.5	0.381
Stature (cm)	177.9 ± 6.6	177 ± 5.4	174.3 ± 5.1	0.099	176 ± 5.4	0.146	177.5 ± 6	0.04 *
BM (kg)	74.5 ± 8.3	73.3 ± 6.5	71.1 ± 6.1	0.159	72.5 ± 6.4	0.146	73.9 ± 7.4	0.079
Sum of SF (mm)	78.5 ± 31	75.6 ± 24.4	77.2 ± 20.6	0.485	76.5 ± 22.9	0.701	77 ± 27.7	0.231
WAnT PP (W) ^a^	1036 ± 138.7	1021.3 ± 148.7	993.5 ± 115.3	0.58	1011.2 ± 136.9	0.318	1028.8 ± 142.9	0.497
WAnT PP/BM (W·kg^−1^) ^b^	14.02 ± 2.13	13.98 ± 2.03	14.04 ± 1.75	0.542	14 ± 1.91	0.267	14 ± 2.06	0.672
WAnT MP (W) ^a^	708.9 ± 67.1	705.5 ± 76.1	684.9 ± 63.5	0.599	698 ± 71.8	0.436	707.2 ± 71.2	0.367
WAnT MP/BM (W·kg^−1^) ^b^	9.62 ± 1.35	9.66 ± 1.1	9.69 ± 1.08	0.49	9.67 ± 1.08	0.674	9.64 ± 1.23	0.232

Values are expressed as the mean ± SD. BM, body mass; SF, skinfolds; WAnT, 30 s Wingate all-out Test; PP, peak power; MP, mean power. * *p* < 0.05. ^a^ Adjusted for age, stature, and BM, ^b^ Adjusted for age.

## Data Availability

The data that support the findings of this study are available upon request from the corresponding author and from the 1000 Genomes Research Study for Iberian Population (Iberian Populations in Spain (IBS)).
